# Study on Performance and Mechanism of SBR and Bio-Oil Recycled SBS Modified Asphalt

**DOI:** 10.3390/polym14235096

**Published:** 2022-11-24

**Authors:** Yuanbo Li, Dongdong Ge, Zihao Ju, Songtao Lv, Yanhua Xue, Yiyang Xue, Liangchen Peng

**Affiliations:** National Engineering Research Center of Highway Maintenance Technology, Changsha University of Science & Technology, Changsha 410114, China

**Keywords:** aged asphalt regeneration, SBR, bio-oil, performance, mechanism

## Abstract

With the continuous development of road construction and maintenance, SBS(Styrene-butadiene-styrene)-modified asphalt is widely used. However, there is no mature method for restoring aged SBS-modified asphalt. This study proposes the use of SBR(polymerized styrene butadiene rubber) and bio-oil for the restoration of aged SBS. In this study, five kinds of recycled asphalt were prepared by adding 5% bio-oil, 10% bio-oil, 6% SBR, 6% SBR + 5% bio-oil, and 6% SBR + 10% bio-oil to long-term aged SBS-modified asphalt. Softening point, penetration, and rotational viscosity experiments were tested to evaluate the conventional properties. Rheological tests revealed the performance of asphalt. Fourier transform infrared spectroscopy (FTIR), and atomic force microscope (AFM) tests were tested to demonstrate the microscopic characteristics of asphalt. Conventional tests investigated that aged asphalt viscosity will increase. Bio-oil could well recycle the asphalt viscosity. SBR could also soften aged asphalt, but its modification effect is limited compared with bio-oil. Rheological tests presented that the SBR and bio-oil have little impact on the temperature sensitivity of SBS-modified asphalt. SBR and bio-oil could decrease the asphalt stiffness. However, SBR and bio-oil could ameliorate the anti-cracking behavior of aged asphalt. The microscopic tests exhibited that SBR and bio-oil could decrease the asphaltene and colloid. Meanwhile, bio-oil could supplement alcohols and ethers at wave number 1000 cm^−1^–1270 cm^−1^. Alcohols and ethers are hard to oxidize, something which has a beneficial role in the anti-aged of recycled asphalt.

## 1. Introduction

With the development of road engineering and the increase of pavement maintenance mileage, asphalt usage increases yearly. As a by-product of petroleum, asphalt is a non-renewable material. With the extensive exploitation of oil resources, petroleum resources will be gradually depleted. At the same time, the demand for road construction and maintenance increased year by year, and the demand for asphalt also increased. During pavement maintenance, the waste aggregate and aged asphalt will pollute the environment [1]. For green transportation and environmental protection development, recycled asphalt has become the focus of research.

SBS-modified asphalt has excellent laboratory performance and durability, so it has been widely applied in the field of road engineering [2]. In the service of asphalt pavement, the ultraviolet rays irradiation [3] and thermal-oxidative aging [4] will lead to the volatilization of light components. Meanwhile, the heavy components will generate in asphalt [5]. After thermal oxidation aging, the penetration, ductility, softening point, and viscosity were decreased [6]. However, the rutting index, strain recovery rate, and stiffness modulus of asphalt were increased. The anti-cracking ability of the asphalt mixture was dramatically decreased, which led to the deterioration of service performance; it will produce many aged SBS-modified asphalt mixtures, so dealing with the regenerated aged SBS-modified asphalt was a significant problem. 

A styrene-butadiene-styrene triple copolymer forms SBS; they create a gel network in the asphalt, strengthening its performance and obtaining good storage stability [7]. On the one hand, SBS polymer gradually breaks the chain and forms small molecules. The performance of SBS-modified asphalt was affected by the aging of SBS-modified asphalt [8]. On the other hand, the light components of SBS-modified asphalt volatilized, and the colloid changed to asphaltene. The asphalt gradually becomes stiff and brittle [9]. The sulfoxide index and aromatic index were significantly increased. During the asphalt aging process, the unsaturated groups break the chemical bond, thus forming polar oxygen-containing functional groups [10]. Long-term aging of SBS-modified asphalt performance seriously degraded, almost brittle in the 5 °C low-temperature ductility test [11]. Scholars have discovered that bio-oil could restore the light components lost in asphalt and have conducted extensive research on bio-oil.

The sulfoxide and aromatic hydrocarbon indexes of bio-oil recycled asphalt remarkably decreased, indicating that bio-oil had a better recovery function for aged asphalt [12]. Lv [13] illustrated the different modification effects of various bio-oil on aged asphalt. The results showed that bio-oil has good compatibility with asphalt binder. From different sources, bio-oil has different regeneration effects on aged asphalt. Bio-oil can supplement the light components in aged asphalt [12], increasing the viscosity and reducing aged asphalt’s stiffness [14]. A.M. [15] found that the consistency and viscosity of modified asphalt with 5% palm oil were consistent with that of the original asphalt. However, the high content of palm oil will weaken the high-temperature rheological behavior of asphalt. De Medeiros Melo Neto [16] found that acidified soybean oil can decrease the temperature sensitivity of aged asphalt. Moreover, it will also defeat the hardness and viscosity of aged asphalt. Bio-oil will decrease the complex modulus (G*) of recycled asphalt [17], and their Jnr will gradually increase as the bio-oil content increases. Girimath [18] tested that when bio-oil content was 2%, 4%, 6%, 8%, and 10%, the Jnr of recycled asphalt binder was 1.64 kPa, 1.65 kPa, 2.56 kPa, 4.91 kPa, and 7.4 kPa, respectively. The results have indicated that bio-oil could negatively affect the anti-rutting properties of the asphalt mixture. Fang [19] used bio-oil such as soybean oil and tung oil to regenerate aged asphalt, and it was found that bio-oil can effectively reduce the aging functional group index. Moreover, it can restore the light components of aged asphalt to an unaged level. At the same time, it can also restore the fluidity of aged asphalt [20].

SBR is characterized by abrasion, heat, and aging resistance, such as SBS; it is a rubber material synthesized from butadiene and styrene as a unit. The cross-linking of rubber can further improve the ductility and anti-aged capability of modified asphalt [21]. SBR-modified asphalt has better ductility and stress relaxation ability [22]. Due to the solubilization reaction between SBR and asphalt, the viscosity will increase when the SBR doping exceeds 2% [23]. Meanwhile, the SBR absorbed the oil in asphalt, which improved the colloidal structure of asphalt. Adding SBR to SBS-modified asphalt also has good compatibility and stability [24]. Ren [25] studied the effect of SBR on aged asphalt. SBR could improve the viscosity, activation energy, temperature sensitivity, and flow resistance of recycled asphalt. With the increase of SBR content, the flow resistance of recycled asphalt gradually increased. Li [26] manifested the stability of cross-linked SBR in different aging periods. The results showed that the cross-linked system remained stable when the aging time was less than 5 h. The viscosity index rapidly increased when the aging time exceeded 5 h, and the cross-linked structure became unstable. SBR-modified asphalt mixture has better anti-fatigue behavior. The durability of the rubber-modified asphalt mixture was improved; its cumulative strain is lower than that of the original mixture. SBR was helpful in the self-healing of cracks [27]. Che [28] utilized SBR to regenerate aged asphalt. Due to the SBR, modified asphalt formed new chemical bonds, which increased the viscosity, G*, G*/sinδ [29]. SBR significantly decreases the creep stiffness of aged asphalt binders. The creep stiffness of aged asphalt with 7% SBR decreased by 79%, indicating that the anti-cracking behavior of recycled asphalt has been ameliorated. Fourier transform infrared spectroscopy (FTIR) results have expressed that new polar functional groups have appeared in recycled asphalt, making SBR-modified asphalt have good elasticity at low temperatures.

In summary, bio-oil was used in the regeneration of aged asphalt. Bio-oil could supplement the light components and enhance the low-temperature behavior of aged asphalt. SBR and SBS have styrene and butadiene copolymer materials, which ameliorate the low-temperature performance. SBR recycled asphalt can also play an excellent anti-fatigue effect; it can provide a new idea for recycled asphalt pavement (RAP). This study conducted laboratory tests on SBR/bio-oil composite regeneration aging SBS-modified asphalt to evaluate its behavior. FTIR and atomic force microscope (AFM) were tested to reveal their microscopic characteristics. This study provides the experimental basis for applying SBR/bio-oil regenerated SBS-modified asphalt.

## 2. Objective

The task of regenerating the widely used SBS-modified asphalt is urgent in order to enhance the sustainable development of resources and the need for ecological and environmental protection [30,31,32]. According to the current theory of SBS-modified asphalt aging mechanism and the comprehensive performance of SBS and bio-oil in the application of regenerated asphalt, we study the transformation of SBS aging from large molecules to small molecules for SBS aging asphalt supplemented with SBR rubber macromolecules. The bio-oil is used to supplement the light components that volatilize and transform into asphaltene [32]. Therefore, it can provide some help for the research of SBS asphalt regeneration.

## 3. Materials and Methods

### 3.1. Material

Table 1 displays the conventional physical performance indexes of SBS-modified asphalt. The test results have shown that the performance indexes of SBS-modified asphalt meet the specification requirement. The SBS-modified asphalt used in this study was produced by Guangzhou Yuande Trading Co., (Guangzhou, China).

SBR and bio-oil were selected as the regenerants. The specific specifications and parameters of SBR are shown in Table 2. SBR is the product of Heng Tai plastic raw materials company (Dongguan, China).

Bio-oil produced by Nantong Yuhao Chemical Technology Co., LTD. (Nantong, China). The specific parameters of bio-oil are shown in Table 3.

### 3.2. Preparation of Recycled Asphalt

First, the SBS-modified asphalt (SBS) was subjected to short-term aging using a Rotating Film Oven (RTFO) at 163 °C for 85 min. Subsequently, the short-term aged asphalt was subjected to long-term aging in a Pressure Aging Vessel (PAV) to obtain SBS long-term aged, modified asphalt (SBS-PAV).

The SBS-PAV was heated continuously for 1 h at 165 °C. First, we prepared the single regenerator-modified asphalt. SBR regenerated asphalt was prepared by blending 6% (asphalt mass ratio) of SBR into SBS-PAV. Bio-oil recycled asphalt was prepared by blending 5% and 10% (asphalt mass ratio) bio-oil into aged SBS-PAV. Next, the SBR was added to bio-oil recycled asphalt to prepare SBR/bio-oil composite recycled asphalt. The bio-oil and SBR contents are shown in Table 4. During the preparation of modified asphalt, the shear rate was 3000 r/min, and the time was 60 min. The temperature was 165 °C during the shear process.

### 3.3. Test Method

#### 3.3.1. Conventional Performance Tests

Softening point, penetration, and viscosity were used to evaluate the conventional properties. Conventional tests ASTM D3461 [33], ASTM D5 [34] and ASTM D4402 [35] tests were conducted.

#### 3.3.2. Dynamic Shear Rheometer Test (DSR)

Temperature sweep (46–88 °C) and Frequency sweep (0.1–10 Hz) tests were conducted. Moreover, the rutting index (G*/sinδ) is used to evaluate the viscoelastic properties of the recycled asphalt. DSR specimens are made with 25 mm molds. The instrument model used in this study is the Anton-Paar MCR302 (Anton Paar, Graz, Austria).

#### 3.3.3. Bending Beam Rheometer Test (BBR)

SBS-PAV and SBS-PAV recycled asphalt were made into 101.6 mm × 12.7 mm × 6.4 mm standard specimens. BBR tests were conducted at −12 °C, −18 °C, −24 °C, and −30 °C. The bending creep stiffness (S) and creep rate (m) were used to describe the anti-creaking behavior of asphalt. According to ASTM D6648-08 [36] specification, S is less than 300 MPa, and m is higher than 0.3. In this study, four samples (SBS original sample, SBS-PAV, 6%SBR + 5%bio, 6%SBR + 10%bio) were prepared for testing at temperatures of −12 °C, −18 °C, −24 °C, and −30 °C. Because 6%SBR + 10%bio recycled asphalt is still very soft at −30 °C, experimental results cannot be obtained.

#### 3.3.4. Fourier Transform Infrared Spectroscopy Test (FTIR)

So as to study the change of functional groups in SBS-PAV caused by regenerant, SBS-PAV and recycled asphalt were performance the FTIR tests. FTIR results can display the functional groups of asphalt. The infrared spectrometer used in this study is Thermo Fisher Nicolet iS50 (Thermo Fisher Scientific, Waltham, MA, USA), with scanning wave numbers of 700–4000 cm^−1^. The instrument has a built-in middle and far infrared diamond ATR module. In this paper, an ATR accessory is used for infrared spectrum analysis.

#### 3.3.5. Atomic Force Microscopy (AFM)

The atomic force microscope (Bruker, Nasdaq, Ettlingen, Germany) uses a microprobe to approach the asphalt surface and generate an interaction force with the asphalt surface. The computer captures the micro force and draws an atomic resolution image of the asphalt surface so that the microsurface morphology and microstructure of the asphalt surface can be observed. The smoothness of the AFM specimen has a large influence on the test results. Therefore, we make the asphalt drops on the silicon sheet, and then the oven is heated for a moment to ensure the smooth surface of the specimen. The atomic force test was carried out at an ambient temperature of 25 °C. The instrument used in this study is Bruker Dimension Icon. This research observes and analyzes SBS, SBS-PAV, 5%bio, 6%SBR, 6%SBR + 5%bio.

## 4. Tests and Results

### 4.1. Conventional Tests

#### 4.1.1. Penetration

**Figure 1:**
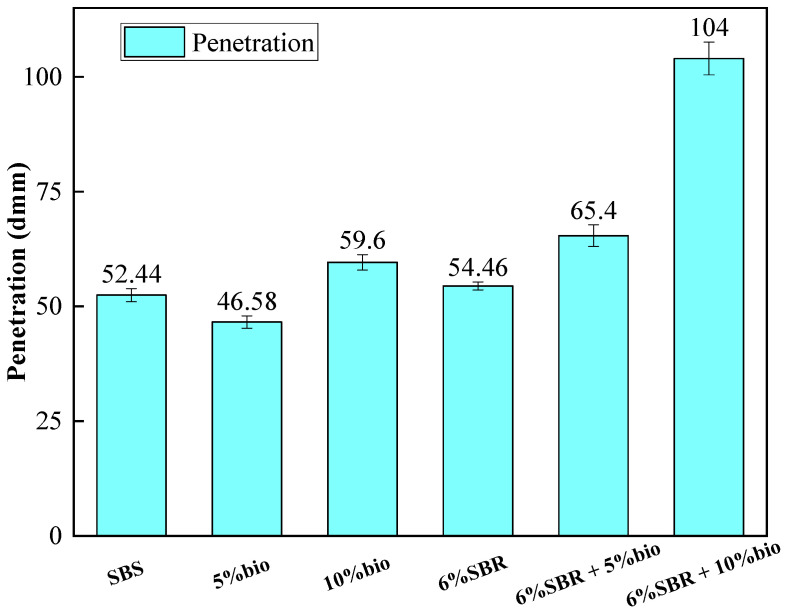
Penetrations of SBS, 5%bio, 10%bio, 6%SBR, 6%SBR + 5%bio and 6%SBR + 10%bio.

The penetrations of SBS and recycled asphalt are shown in Figure 1. The penetration of 5%bio is 21.8% lower than 10%bio, while the penetration of 10%bio has exceeded that of SBS by 13%. 6%SBR + 5%bio can make the penetration of asphalt recovery. 6%SBR + 5%bio had 20% higher penetration than 6%SBR alone. The penetration of 6%SBR + 10% bio is nearly twice as high as that of 6%SBR. The increase in penetration is due to the fact that bio-oil contains light components similar to asphalt, and the content of light components is closely related to the penetration of asphalt [37]. The combination of bio-oil and SBR will substantially increase the penetration of aged asphalt.

#### 4.1.2. Softening Point

**Figure 2:**
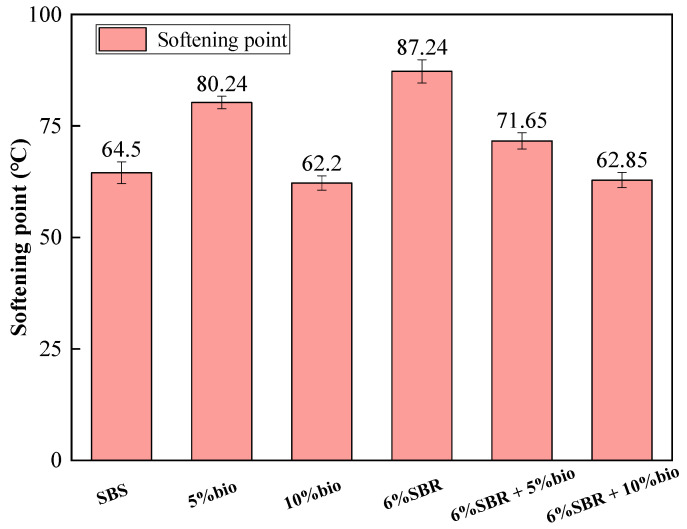
Softening points of SBS, 5%bio, 10%bio, 6%SBR, 6%SBR + 5%bio and 6%SBR + 10%bio.

The softening point of SBS and recycled asphalt are shown in Figure 2. The softening point of 6%SBR is 35.3% higher than SBS, and 6%SBR had limited ability to recover the softening point of long-term aging asphalt. When the bio-oil is added at 5%, the softening point is 80.24 °C. When the incorporation of bio-oil was increased to 10%, the softening point was 62.2 °C, which decreased by 19.6%. Bio-oil alone has some effect on the high-temperature performance of asphalt. The softening point of 6%SBR was 22.74 °C higher compared to SBS. However, the recovery of high-temperature sensitivity of SBR to aging asphalt was more limited. The softening point of 6%SBR + 10%bio was 14% lower compared to 6%SBR + 5%bio. The core substance of the asphalt colloid system is asphaltene, which has high content and a high softening point [38]. When bio-oil supplements the light components of aged asphalt, the proportion of asphaltene also decreases. The softening point of recycled asphalt decreases with the incorporation of bio-oil blending.

#### 4.1.3. Viscosity

**Figure 3:**
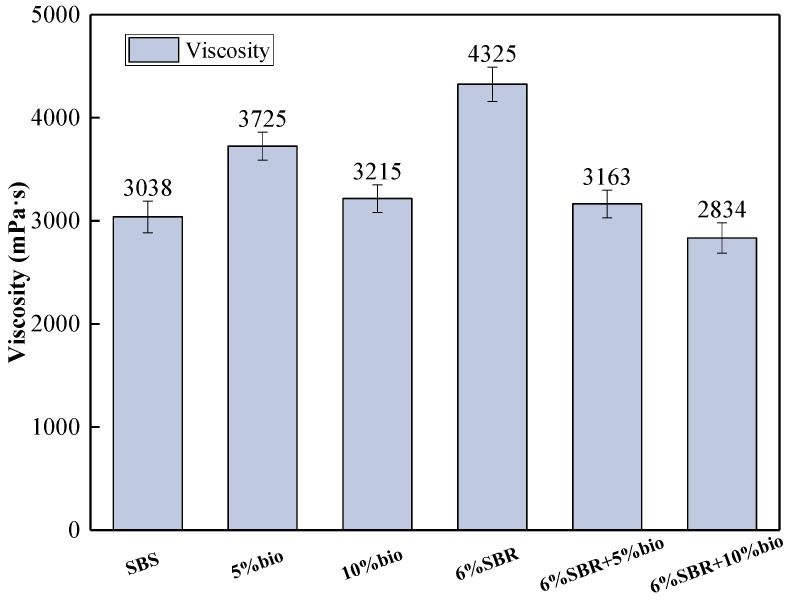
Viscosities of SBS, 5%bio, 10%bio, 6%SBR, 6%SBR + 5%bio and 6%SBR + 10%bio.

The viscosity of SBS and recycled asphalt at 135 °C are shown in Figure 3. The viscosity of 6%SBR was higher than SBS by 42.4%; its viscosity is higher, and the viscosity of 6%SBR is too high. The energy consumption of pavement construction will increase accordingly. 10%bio has a lower viscosity compared to 5%bio, and the viscosity is reduced by 15.9%. Mixing bio-oil alone will make the viscosity of long-term aging asphalt lower. When SBR and bio-oil were compounded in recycled asphalt, the viscosity of recycled asphalt decreased with the incorporation of bio-oil. However, the viscosity in 6%SBR + 10%bio was lower than 3000 mpa·s, which did not comply with the specification, while the viscosity of 6%SBR + 5%bio was closest to that of SBS, and the viscosity recovery was better. The saturated and aromatic fractions in the asphalt have less effect on the viscosity of asphalt. Asphaltene has an influence on viscosity, while bio-oil supplements the light components and reduces the percentage of asphaltene, thus indirectly reducing the viscosity of asphalt. The reduction of viscosity is conducive to improving the workability of asphalt pavement.

### 4.2. Rheological Tests

#### 4.2.1. Temperature Sweep

**Figure 4:**
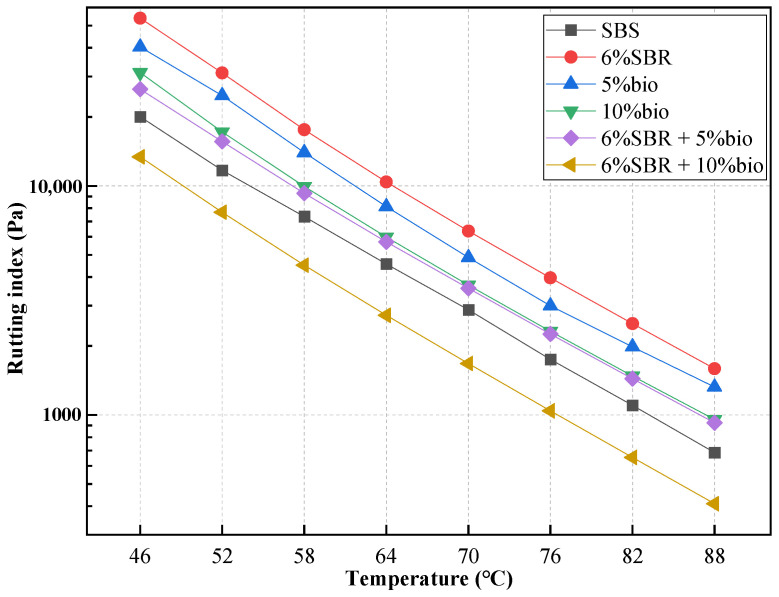
Temperature sweep of SBS, 5%bio, 10%bio, 6%SBR, 6%SBR + 5%bio and 6%SBR + 10%bio.

In this study, temperature sweep tests were conducted on SBS, 5%bio, 10%bio, 6%SBR, 6%SBR + 5%bio, and 6%SBR + 10%bio. The rutting index is an evaluation index to assess the high-temperature performance of different asphalts. The G*/sinδ value is shown in Figure 4.

At 64 °C, the G*/sinδ with 5%bio was 8114 Pa. Moreover, G*/sinδ with 10%bio was 5972 Pa, which decreased by 26.4% compared with 5%bio. With the bio-oil content increase, the G*/sinδ process of recycled asphalt decreased, and 5%bio had better rutting resistance than that of 10%bio. However, the G*/sinδ of 6%SBR at 64 °C is 10393 Pa, which was much higher than SBS 4563 Pa and bio-oil recycled asphalt. 6%SBR has the highest resistance to rutting deformation. Compared with the recycling effect of bio-oil and SBR, SBR has less recovery ability to rutting index than that of bio-oil.

The 6%SBR + 5%bio is 24.8% higher than SBS recycled asphalt’s rutting index, and its G*/sinδ is 5695 Pa. The combined effect of SBS and bio-oil is more obvious than that of bio-oil and SBR alone, and its G*/sinδ is lower than that of 6%SBR and 5%bio. From the index, it is the decline of resistance rutting ability, and from the recovery of asphalt, it is better for the recovery effect of aging asphalt.

The G*/sinδ of 6%SBR + 10%bio is 52.2% lower than 6%SBR + 5%bio; its G*/sinδ is 2722 Pa. Under the joint action of SBR and bio-oil, its G*/sinδ is far lower than that of SBS. The rutting resistance of 6%SBR + 10%bio could not meet road engineering.

Comparing SBR and bio-oil alone, SBR and bio-oil alone could restore the rutting resistance of aged asphalt to some extent. Under the coupling effect of SBR and bio-oil, composite restoring could restore the rutting index to a large extent, but the excessive amount of padding would make the asphalt not meet the requirement of road engineering.

#### 4.2.2. Frequency Sweep

**Figure 5:**
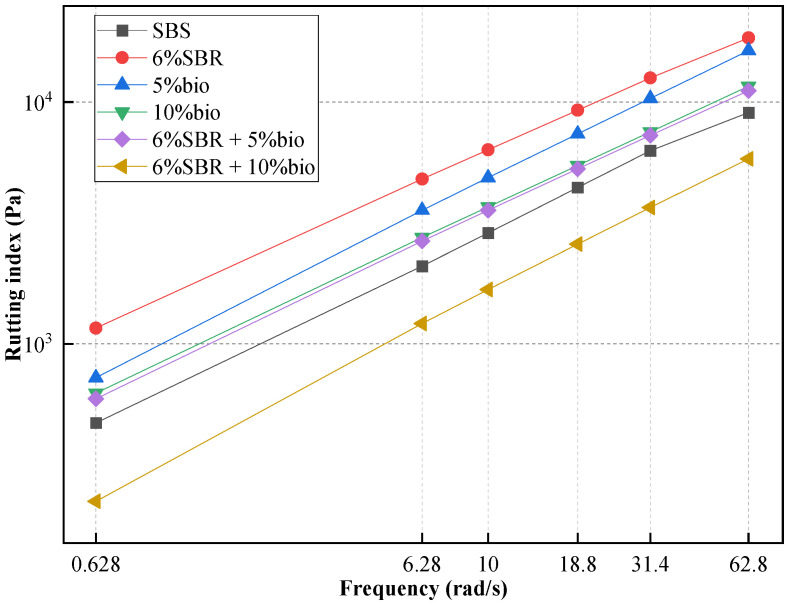
Frequency sweep (70 °C) of SBS, 5%bio, 10%bio, 6%SBR, 6%SBR + 5%bio and 6%SBR + 10%bio.

**Figure 6:**
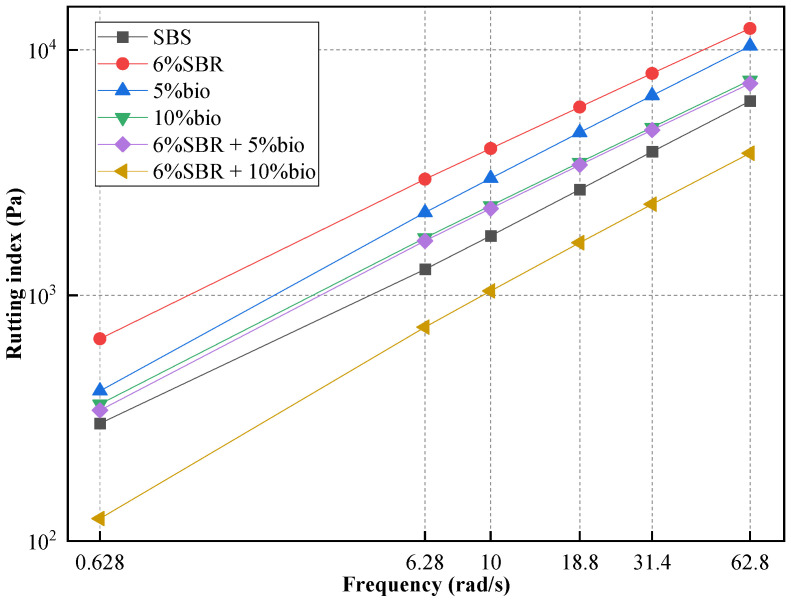
Frequency sweep (76 °C) of SBS, 5%bio, 10%bio, 6%SBR, 6%SBR + 5%bio and 6%SBR + 10%bio.

A frequency sweep test of SBS and recycled asphalt was performed at 70 °C and 76 °C, and the test results are shown in Figure 5 and Figure 6. Frequency sweep test results could reflect the frequency sensitivity of asphalt. With the increase in frequency, asphalt’s rutting index gradually increased. This shows that rutting deformation is not easy to occur on asphalt road surfaces under high-speed driving. In Figure 5 and Figure 6, the Frequency sweep test results in recycled asphalt are basically consistent with the changing trend of SBS, indicating that the frequency sensitivity of recycled asphalt and SBS is the same. 

As shown in Figure 5, when the loading frequency is 10 rad/s, the maximum rutting index of 6%SBR was 6352.8 Pa, which increased by 120.76% compared with SBS. This shows that 6%SBR has higher elastic properties and higher asphalt stiffness. However, the rutting index of 5%bio and 10%bio decreased significantly compared with 6%SBR, which was closer to SBS. The rutting index at 70 °C at the loading frequency of 10 rad/s and 5%bio and 10%bio are 7389.1 Pa and 5468.6 Pa. Compared with SBS asphalt, the G*/sinδ of 5%bio and 10% bio increased by 68.5% and 28.59%. With the addition of bio-oil content, the G*/sinδ decreased obviously. The single addition of 6%SBR and 5%bio could not restore the high-temperature performance of aged SBS to its original state. When SBR and bio-oil are combined, we can find that the rutting index in recycled asphalt has obviously decreased. Figure 5 and Figure 6 both show that the rutting index of 6%SBR + 5%bio was basically the same as that of 10%bio.

The rutting index of 6%SBR + 10%bio was inferior to SBS, and its high-temperature performance was reduced. However, the rutting index of 6%SBR + 5%bio was slightly higher than SBS, which indicates that 6% SBR and 5% bio-oil could basically restore the aging SBS to its original state. Among them, bio-oil could supplement the light components volatilized by asphalt during aging [12]. However, SBR would supplement SBS cleaved during aging and endow recycled asphalt with elastic recovery performance [28].

#### 4.2.3. Bending Beam Rheometer Test (BBR)

To study the low-temperature crack resistance of asphalt, BBR tests were carried out on SBS, SBS-PAV, and 6%SBR + 5%bio. The greater the stiffness modulus (S) obtained from the test. The stiffer and more brittle the asphalt is at low temperatures. The creep rate (m) reflects the rate at which the stiffness modulus changes with time. The larger m is, the better the asphalt’s dissipation capacity for temperature stress. At the same time, ASTM D6648 requires that the m value should be greater than 0.3 and the S value should be less than 300 MPa. Table 5 displays the BBR results of modifier asphalt.

In Table 5, the S value of SBS is 202 MPa, and the m value is 0.318 at −18 °C. The low-temperature rheology of SBS meets the standard requirements. The results show that SBS has great low-temperature crack resistance at −18 °C, and SBS has excellent thermal stress dissipation capacity. After PAV, under the same temperature of −18 °C, the S value of SBS-PAV asphalt is 316 MPa, and the m value is 0.273, which do not meet the specification requirements, indicating that the asphalt after aging has a large stiffness at low temperature, and its low-temperature crack resistance is greatly reduced. The S value of 6%SBR + 5%bio obtained from the combined regeneration of SBR and bio-oil is 67.4 MPa, and the m value is 0.435 measured at −18 °C, which not only meets the specification requirements but also has better low-temperature crack resistance compared with the SBS; it can be seen from the data in Table 5 that under the environment of −24 °C, 6%SBR + 5%bio still meet the specification requirements, but SBS asphalt does not; it shows that SBR and bio-oil composite regeneration can heighten the low-temperature toughness of asphalt, which is conducive to restoring the low-temperature crack resistance of aged asphalt. SBR rubber has good low-temperature resistance due to the presence of butadiene chains, which are blended in asphalt to form a mesh structure to help enhance the low-temperature performance of asphalt.

### 4.3. Microscopic Test

#### 4.3.1. Fourier Transform Infrared Spectroscopy Test (FTIR)

**Figure 7:**
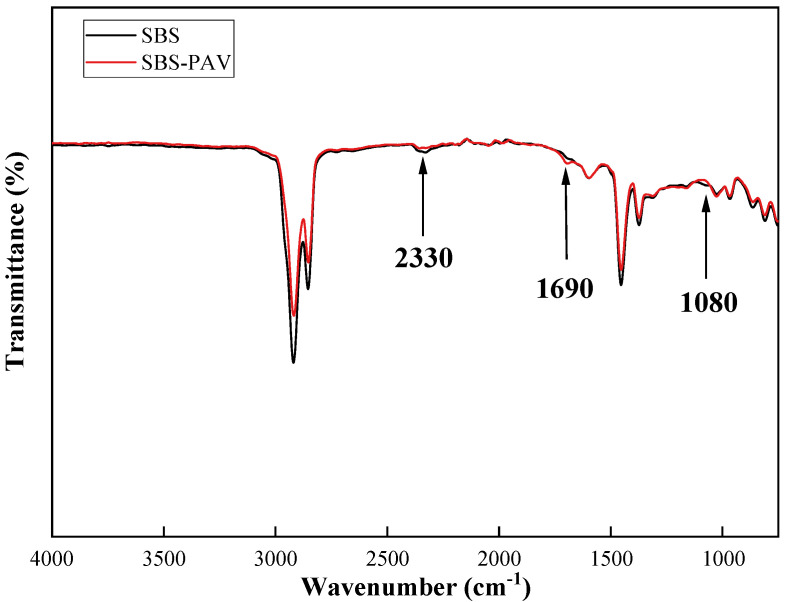
Infrared spectrum of SBS and SBS-PAV.

The SBS, SBS-PAV, 6%SBR, 5%bio, and 6%SBR + 5%bio were tested by the infrared spectrometer, and the aging mechanism and regeneration mechanism were analyzed from the perspective of chemical functional groups. Figure 7 is the infrared spectrum comparison diagram of SBS and SBS-PAV.

By analyzing the FTIR spectra of SBS and SBS-PAV, we can find subtle differences in the chemical composition of the asphalt during long-term aging in Figure 7. Their plots are incredibly similar. However, there are still some peaks of increase and contraction in SBS-modified asphalt after aging. 2330 cm^−1^ corresponds to the C=O stretching vibration of CO_2_, which may be caused by the CO_2_ in the air during the test [6]. There is a peak increase in SBS-PAV at wave number 1690 cm^−1^; 1690 cm^−1^ corresponds to C=C stretching vibration and C-O stretching vibration in olefins or aromatics; it is caused by the absorption of oxygen by unsaturated carbon chains, while C=C is mainly from SBS. The contraction of the peak at 1080 cm^−1^ is caused by the reaction of the elemental sulfur present in the asphalt with oxygen. Firstly, the increase of the polar covalent bond C-O and the decrease of the nonpolar covalent bond C-H lead to the increase of the bitumen viscosity. Second, SBS contains polybutadiene segments and polystyrene chains. Chain segments containing C=C aging accelerate the decomposition of SBS. The degradation of SBS is accelerated by aging, which indicates that aging has a greater effect on light components such as olefins or aromatics.

**Figure 8:**
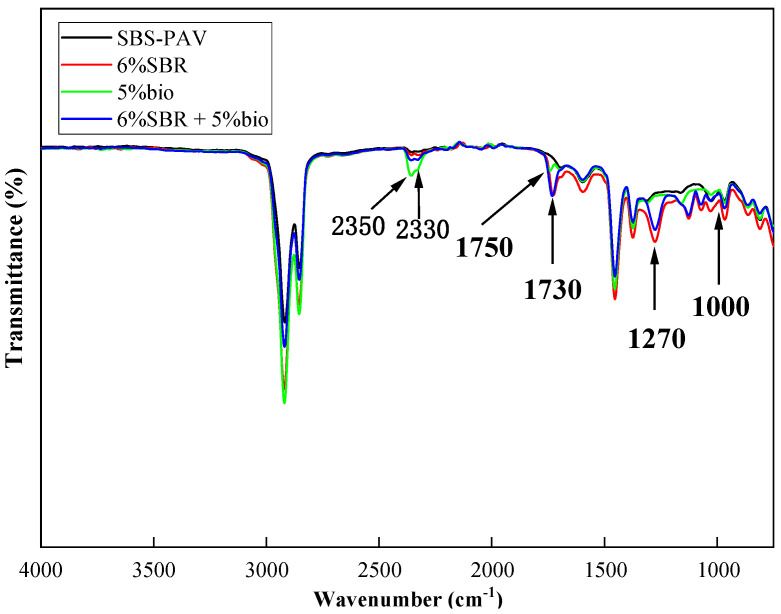
Infrared spectra of SBS-PAV, 6%SBR, 5%bio and 6%SBR+5%bio.

Figure 8 shows the FTIR spectra of SBS-PAV and 6%SBR, 5%bio, and 6%SBR + 5%bio. 5%bio shows a new peak at 1750 cm^−1^. There are multiple groups superimposed near the 1750 cm^−1^ wave number, including aldehyde groups, ester groups, carboxyl groups, and other groups. We first excluded aldehyde groups because they must have moderate peaks at 1400 and 1100 cm^−1^, and the peak at 1750 cm^−1^ must be a strong signal. Finally, the ester group was identified because 5%bio showed a new peak at 1160 cm^−1^, which belongs to the signal of the ester group vibration. The bio-oil produced a C=O stretching vibration at 1750 cm^−1^. A superposition peak appears at 2330 cm^−1^ with wave number attributed to C=O stretching vibration due to the incorporation of SBR. The absorption peak at wave number 1270 cm^−1^, specific to SBR, is the stretching vibration of the olefin C-H bond and carboxyl C-O bond. The absorption peaks at 1000–1270 cm^−1^ represent the C-O stretching vibration of ether and carboxyl groups. However, the others are not easily oxidized, which has a good effect on the aging resistance of recycled asphalt. The weak absorption peak near 960 cm^−1^ is caused by the stretching vibration of C-H. the absorption peak at 2330–2350 cm^−1^ corresponds to the C=O vibration of CO_2_, which may be caused by the action of CO_2_ in the air during the test [6].

#### 4.3.2. Atomic Force Microscope (AFM)

**Figure 9:**
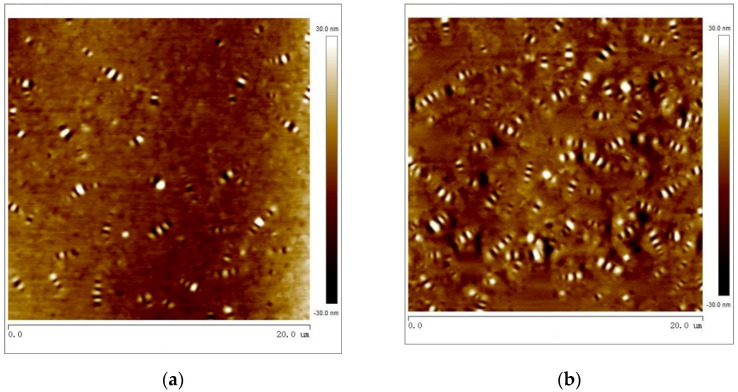

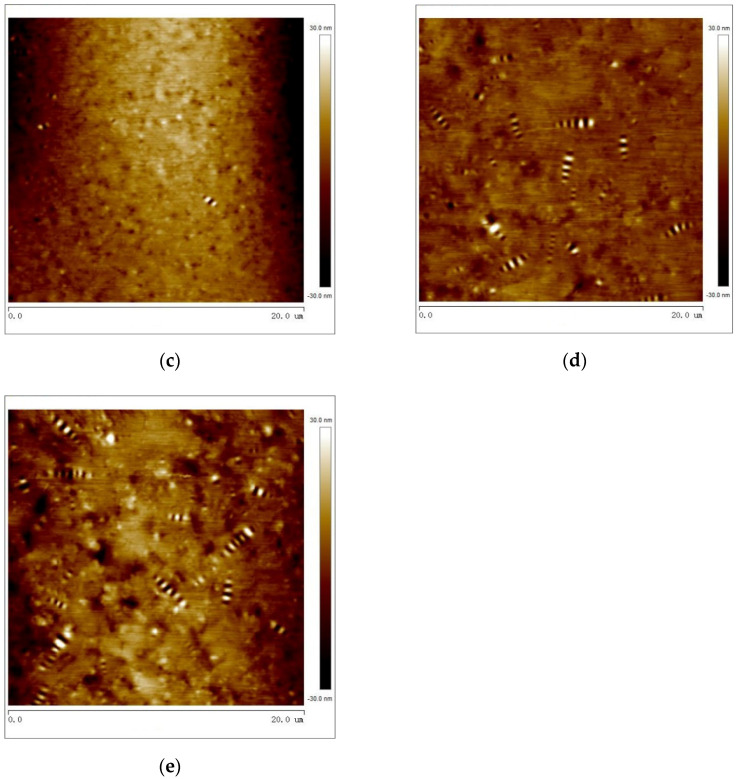
Microstructures of (**a**) SBS (**b**) SBS-PAV (**c**) 5%bio (**d**) 6%SBR (**e**) 6%SBR + 5%bio.

The SBS, SBS-PAV, 5%bio, 6%SBR, and 6%SBR + 5%bio morphologies observed by AFM scan are shown in Figure 9. The average lengths of the bee structures are shown in Table 6.

In this study, NanoScope Analysis software was used to analyze the average length and average area of the bee structures. The area of individual bee structures has increased by 134% after PAV aging, and the average increase in length of individual bee structures after PAV aging is 15% from 2.24 nm to 2.58 nm. The area increases from 0.81 μm^2^ to 0.96 μm^2^. Figure 9c,d shows that length and area are significantly smaller. Compared with PAV long-term aged asphalt, the bee structure in 5%bio is shortened by 38%, and the area of single bee structure is reduced by 89%. Compared with PAV long-term aged asphalt, the bee structure in 6%SBR is shortened by 24%, and the area of single bee structure is reduced by 25%. The average area of a single bee structure can visually reflect the adsorption of the asphalt colloidal structure to the components. The change in the average area of single bee structure before and after aging can be used as an evaluation index of asphalt aging degree [39]. Because the bee structure is mainly wax crystalline colloid and asphaltene, the light-yellow part is mainly composed of aromatic phenol, saturated phenol, and other light components. The blending of bio-oil and SBR supplements the light components in SBS-PAV, which makes the distance between asphaltene and colloid in the light components far away and reduces the aggregation of asphaltene.

In Table 7, the arithmetic mean roughness R_a_ and root-mean-square roughness R_q_ of the SBS as-built samples are greater than that of SBS-PAV. SBS-PAV roughness is 31% and 35% lower than the original SBS asphalt R_a_ and R_q_, respectively. There is a correlation between lower roughness and more and larger bee structures. Thermal oxidative aging reduces the surface roughness of the asphalt and reduces the surface energy of the asphalt, which in turn has an effect on aggregate adhesion. The roughness R_a_ and R_q_ of 6%SBR + 5%bio are restored by 96% and 82%, respectively, relative to the original SBS asphalt. The above data reveal that SBR and bio-oil supplemented the lighter fraction of asphalt during the regeneration process, and the ratio of asphaltene to gum was reduced. Moreover, the light component has a restraint effect on the wax crystallization of the bee structure, and the peak of the bee structure is reduced, thus reducing the roughness.

DMT modulus is Young’s modulus calculated by fitting the force curve with the Derjaguin–Muller–Toropov model. Table 8 shows the measured adhesion and Young’s modulus of SBS, SBS-PAV, 5%bio, 6%SBR, and 6%SBR + 5%bio.

Table 8 shows that the adhesion of SBS-PAV decreases by 47% after long-term aging, Young’s modulus of DMT increased by 24%, and asphaltene and colloid of asphalt after PAV aging increased. When 5% bio was added, Young’s modulus of 5% bio decreased by 49%. Macroscopically, asphalt softened, penetration increased, and green softened. In dry room temperature, the order of adhesion is SBS > 6%SBR > 6%SBR + 5%bio > SBS-PAV > 5%bio. The adhesion of unaged SBS is higher than that of aged asphalt and recycled asphalt. Still, the adhesion of bio-oil and SBR co-recycled asphalt is higher than that of SBS-PAV, and the adhesion of bio-oil and SBR co-recycled asphalt to aggregate is restored.

## 5. Discussions and Conclusions

SBS was regenerated and aged by mixing 6%SBR and different proportions (5% and 10%) of bio-oil. Conventional properties and rheological properties tests were used to reveal the properties of recycled asphalt. FTIR and AFM experiments were carried out to reveal the regeneration mechanism. The following are the main conclusions of this study.

Under the combined action of SBR and bio-oil, the penetration of aged asphalt was restored. The workability of asphalt regenerated by bio-oil and SBR was improved. Under the effects of 6% SBR and 10% bio-oil, the penetration far exceeds that of SBS. At the same time, the viscosity was obviously reduced.

DSR and BBR tests proved the rheological behavior of recycled asphalt. SBS asphalt after PAV had higher stiffness, and the recovery effect of SBR and bio-oil regenerated asphalt was not enough. Bio-oil has a better viscosity reduction effect and can enhance the anti-creaking behavior of asphalt, but it will weaken the asphalt’s high-temperature behavior. SBR has a general viscosity reduction effect. Adding SBR and bio-oil together in aged asphalt could play their advantages, and the regeneration effect is best.

AFM analysis has shown that bio-oil could soften asphalt, supplementing light components of SBS-PAV. SBR could restore the polymer in asphalt, thus increasing the adhesion and roughness of asphalt, positively impacting the fatigue resistance of the asphalt mixture. The new functional groups in recycled asphalt were known that bio-oil and SBR mainly supplemented with aromatic and polar functional groups at wave numbers of 1000–1270 cm^−1^. These light components maintain good elasticity at low temperatures; they make the composite recycled asphalt of SBR and bio-oil have good anti-creaking behavior.

In this study, we have contributed to the restoration of SBS-modified asphalt aged with SBR and bio-oil composite by conventional, rheological and microscopic tests on the recycled asphalt. In the next step, we will investigate the effects of various bio-oils and their component differences on the macroscopic properties of recycled asphalt.

## Figures and Tables

**Table 1:** Properties of SBS modified asphalt.

Properties	Unit	Measured Result	Requirements
Penetration	0.1 mm	54.5	40–60
Softening point	°C	85.9	≥60
Ductility (5 °C)	cm	38.8	≥20
Flash point	°C	261	≥230
Weight loss after RTFO	%	0.022	≤±1.0
Residual penetration ratio after RTFO	%	75.7	≥65
Residual ductility after RTFO (5 °C)	cm	24.2	≥15

**Table 2:** Properties of SBR modifier.

Properties	Unit	Results
PH	/	7~9
Viscosity	mpa·s	50~80
tensile strength at 145 °C	MPa	≥22
Styrene content	/	22~28%

**Table 3:** Properties of bio-oil.

Properties	Unit	Results
Viscosity (60 °C)	Pa·s	75
Flash point	°C	232
Density (15 °C)	g/mL	0.94

**Table 4:** Sample preparation table.

SBR Content	Bio-Oil Content	Abbreviation
0%	0	SBS
	5%	5%bio
	10%	10%bio
6%	0%	6%SBR
	5%	6%SBR + 5%bio
	10%	6%SBR + 10%bio

**Table 5:** BBR test results of modified asphalt.

Asphalt Types	Test Condition	m-Value	S/MPa
SBS	−18 °C	0.318	202
	−24 °C	0.24	435
SBS-PAV	−12 °C	0.346	133
	−18 °C	0.273	316
6%SBR + 5%bio	−18 °C	0.435	67.4
	−24 °C	0.359	177
	−30 °C	0.275	414

**Table 6:** Bee structure’s average length of SBS and modified asphalt.

Asphalt Types	Average Length of Bee Structures (μm)
SBS	2.24
SBS-PAV	2.58
5%bio	0.92
6%SBR	1.96
6%SBR + 5%bio	2.22

**Table 7:** The roughness of SBS and modified asphalt.

Roughness Evaluation Index	R_a_ (nm)	R_q_ (nm)
SBS	4.61	7.07
SBS-PAV	3.16	4.63
5%bio	2.86	3.63
6%SBR	3.47	4.22
6%SBR + 5%bio	4.43	5.83

**Table 8:** Adhesion and DMT modulus of SBS and modified asphalt.

Asphalt Types	Adhesion (nN)	DMT Modulus (MPa)
SBS	18.2	489
PAV	9.5	608
5%bio	6.6	310
6%SBR	15.3	508
6%SBR + 5%bio	13.8	285

## Data Availability

Not applicable.
